# Inhibitory Effect of Sauchinone on UDP-Glucuronosyltransferase (UGT) 2B7 Activity

**DOI:** 10.3390/molecules23020366

**Published:** 2018-02-09

**Authors:** Byoung Hoon You, Eun Chae Gong, Young Hee Choi

**Affiliations:** College of Pharmacy and Intergrated Research Institute for Drug Development, Dongguk University-Seoul, 32 Dongguk-lo, Ilsandong-gu, Goyang, Gyonggi-do 10326, Korea; hoon4131@nate.com (B.H.Y.); dmsco3901@hanmail.net (E.C.G.)

**Keywords:** sauchinone, *Saururus chinensis*, UGT2B7, inhibition, drug interaction

## Abstract

Herb–drug interaction (HDI) limits clinical application of herbs and drugs, and inhibition of herbs towards uridine diphosphate (UDP)-glucuronosyltransferases (UGTs) has gained attention as one of the important reasons to cause HDIs. Sauchinone, an active lignan isolated from aerial parts of *Saururus chinensis* (Saururacease), possesses anti-oxidant, anti-inflammatory, and anti-viral activities. In pharmacokinetics of sauchinone, sauchinone is highly distributed to the liver, forming extensive metabolites of sauchinone via UGTs in the liver. Thus, we investigated whether sauchinone inhibited UGTs to explore potential of sauchinone–drug interactions. In human liver microsomes (HLMs), sauchinone inhibited activities of UGT1A1, 1A3, 1A6, and 2B7 with IC_50_ values of 8.83, 43.9, 0.758, and 0.279 μM, respectively. Sauchinone also noncompetitively inhibited UGT1A6 and 2B7 with *K*_i_ values of 1.08 and 0.524 μM, respectively. In in vivo interaction study using mice, sauchinone inhibited UGT2B7-mediated zidovudine metabolism, resulting in increased systemic exposure of zidovudine when sauchinone and zidovudine were co-administered together. Our results indicated that there is potential HDI between sauchinone and drugs undergoing UGT2B7-mediated metabolism, possibly contributing to the safe use of sauchinone and drug combinations.

## 1. Introduction

Herb–drug combinations have been steadily increased and encouraged as main medical treatments by the World Health Organization [1,2]. Since herb–drug interactions (HDIs) can limit clinical application of herbs and drugs, inhibition of herbs toward uridine diphosphate (UDP)-glucuronosyltransferases (UGTs) has received attention in association with alternations of drug efficacy or toxicity [3,4,5]. 

UGTs are phase II metabolic enzymes that predominantly catalyze glucuronidation of xenobiotics, including approximately 35% of drugs and consequently, facilitating elimination of glucuronidated metabolites through bile and urine [6,7]. Glucuronidation clears drugs because glucuronidated metabolites have more polarity and water solubility. It also detoxifies drugs because glucuronidated metabolites possess less activity or toxicity than their parent forms [8]. Many cases of UGT inhibition-mediated drug interactions have been reported [5,7] including UGT1A1 inhibition by psoralidin that causes irinotecan’ toxicity [9], UGT1A3 inhibition by gemfibrozil that enhances susceptibility of statins [10], UGT1A6 inhibition by silybin that attenuates fenofibrate-induced UGT1A6 [11], UGT1A6 inhibition by phenobarbital and phenytoin that causes hepatotoxicity of acetamoniphen [12,13], UGT1A9 inhibition by mefenamic acid that changes exposure of dapagliflozin’s metabolite [14], and UGT2B7 inhibition by valproic acid that changes efficacy and toxicity of zidovudine [15]. Regulatory agencies have recognized the importance of UGT-mediated drug interactions in drug discovery research and patients’ safety [2,16,17]. Moreover, the U.S. FDA strongly recommends the evaluation of UGT-mediated interactions in HDIs [3,16,17,18].

*Saururus chinensis* Hort. ex Loudon (Saururaceae), commonly known as Chinese lizard’s tail or Sam-baek-cho in Korea, is a plant with a long history of medical use. It has been used to treat hepatitis in Korea [19,20] and edema, pneumonia, jaundice, gonorrhea and inflammatory disease in North Asia [21]. Sauchinone is a biologically active lignan isolated from aerial parts of *S. chinensis*, showing anti-inflammatory and anti-viral activities [22,23]. In pharmacokinetic studies, sauchinone is highly distributed to the liver where UGTs can extensively catalyze sauchinone metabolism [24,25]. Thus, there might be potential of sauchinone–drug interaction through UGT-mediated metabolism. However, currently there is no information regarding inhibition of UGTs by sauchinone. Therefore, the objective of this study was to determine the inhibitory effect of sauchinone on UGT1A1, 1A3, 1A4, 1A6, 1A9, and 2B7 activities. Inhibition kinetics of sauchinone was then investigated in in vitro using human liver microsomes (HLMs). Pharmacokinetic interaction of sauchinone and zidovudine, a substrate of UGT2B7, was also evaluated in vivo using mice.

## 2. Results

### 2.1. Inhibitory Effects of Sauchinone on UGT Activities

**Figure 1:**
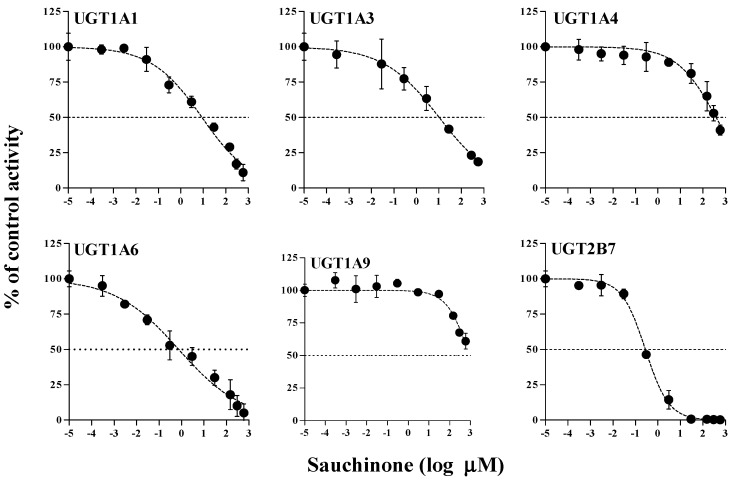
IC_50_ curves for the direct inhibition of UGT1A1, UGT1A3, UGT1A4, UGT1A6, UGT1A9, and UGT2B7 in HLMs. The ‘●’ represents the remaining percentage of UGT-mediated metabolic activity with sauchinone as an inhibitor versus control (without sauchinone). (*n* = 3 for each group).

The inhibitory effects of sauchinone on UGT1A1, 1A3, 1A4, 1A6, 1A9, and 2B7 were shown in Figure 1 and their IC_50_ values are listed in Table 1. When sauchinone concentrations were increased to 200 μM, IC_50_ values of UGT1A1-catalyzed β-estradiol, UGT1A3-catalyzed CDCA, 1A6-catalyzed serotonin, and 2B7-catalyzed zidovudine (8.83, 43.9, 0.758, and 0.279 μM, respectively) were lower than those of well-known inhibitors such as chrysin, lithocholic acid, 1-naphthol, and efavirenz (28.3, 69.8, 35.2, and 75.4 μM respectively), indicating that sauchione inhibited metabolic activities of UGT1A1, 1A3, 1A6, and 2B7 in HLM. Sauchinone also showed negligible inhibitory effect on UGT1A4 and 1A9 activities under our study conditions.

### 2.2. K_i_ of Sauchinone on UGT1A6 and 2B7-Mediated Glucuronidation Activities

**Figure 2:**
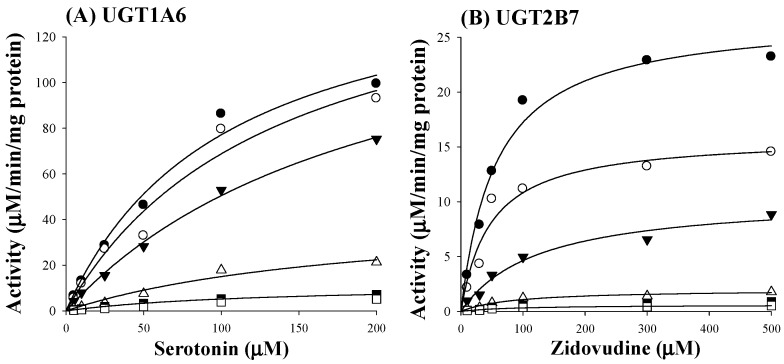

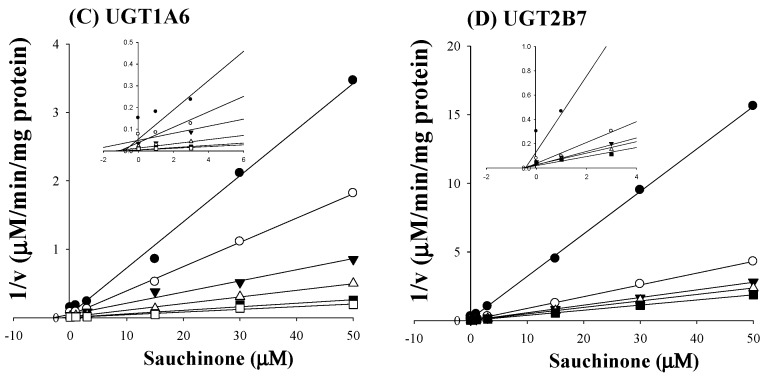
Inhibition of UGT1A6 and 2B7 by sauchinone. (**A**) The plot for velocities of UGT1A6-mediated serotonin glucuronidation versus serotonin concentrations depending on sauchinone concentrations. The following symbols (●, ○, ▼, △, ■, □) represent sauchinonne (as an inhibitor) concentrations of 0, 1, 3, 15, 30, and 50 μM, respectively; (**B**) The plot for velocities of UGT2B7-mediated zidovudine glucuronidation versus zidovudine concentrations depending on sauchinone concentrations. The following symbols (●, ○, ▼, △, ■, □) represent sauchinonne (as an inhibitor) concentrations of 0, 1, 3, 15, 30, and 50 μM, respectively; (**C**) Dixon plot of inhibitory effect of sauchinone on UGT1A6-mediated serotonin glucuronidation activity. The following symbols (●, ○, ▼, △, ■, □) represent sauchinonne (as an inhibitor) concentrations of 0, 1, 3, 15, 30, and 50 μM, respectively; (**D**) Dixon plot of inhibitory effect of sauchinone on UGT2B7-mediated zidovudine glucuronidation activity. The following symbols (●, ○, ▼, △, ■, □) represent sauchinonne (as an inhibitor) concentrations of 0, 1, 3, 15, 30, and 50 μM, respectively.

Based on estimated IC_50_ values of sauchinone, enzyme kinetic assays of sauchinone on inhibition of UGT1A6 and 2B7 activities were conducted. Various concentrations of sauchinone were used and *K*_i_ values of sauchinone for UGT1A6 and 2B7 were estimated. Figure 2A,B showed the activities of UGT1A6-mediated serotonin glucuronidation and UGT2B7-mediated zidovudine glucuronidation with and without sauchinone as an inhibitor using nonlinear regression analysis. In Figure 2A, the maximum rate of metabolic activity (V_max_) and Michaelis-Menten constant (K_m_, the substrate concentration at which the reaction rate is half of V_max_) for UGT1A6-mediated serotonin glucuronidation without sauchinone were 174 μM/min/mg protein and 126 μM, respectively. As increasing sauchinone (as an inhibitor) concentrations from 0 to 50 μM, V_max_ tended to decrease from 174 to 6.36 μM/min/mg protein, but K_m_ represented in the ranges of 103–126 μM for UGT1A6-mediated serotonin glucuronidation. Also in Figure 2B, V_max_ and K_m_ for UGT2B7-mediated zidovudine glucuronidation without sauchinone were 31.5 μM/min/mg protein and 85 μM, respectively. With increasing sauchinone concentrations (as an inhibitor) from 0 to 50 μM, V_max_ tended to decrease from 31.5 to 0.495 μM/min/mg protein, but K_m_ represented in the ranges of 70.2–85.0 μM for UGT2B7-mediated zidovudine glucuronidation.

As shown in Figure 2C,D, all Dixon plots for the inhibition of sauchinone on UGT1A6 and 2B7 fitted well with the noncompetitive inhibition mode in visual inspection [25], suggesting that sauchinone could noncompetitively inhibit UGT1A6 and 2B7. *K*_i_ values of sauchinone for UGT1A6 and 2B7 were 1.08 (0.811–1.37) μM and 0.524 (0.442–0.610) μM, respectively. 

### 2.3. Inhibitory Effects of Sauchinone on Ugt Activities Using Mice Liver Microsomes (MLMs)

**Figure 3:**
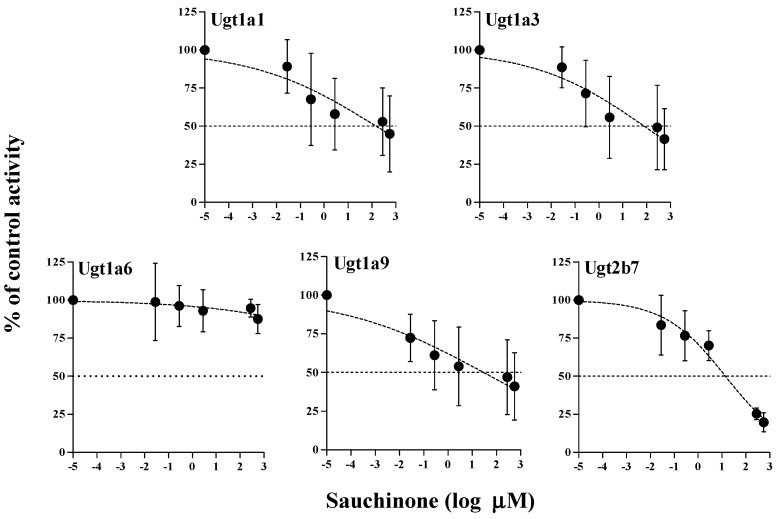
IC_50_ curves for the direct inhibition of Ugt1a1, Ugt1a3, Ugt1a6, Ugt1a9, and Ugt2b7 in MLM. The ‘●’ represents the remaining percentage of UGT-mediated metabolic activity with sauchinone as an inhibitor versus control (without sauchinone). (*n* = 3 for each group).

The inhibitory effects of sauchinone on Ugt1a1, 1a3, 1a6, 1a9, and 2b7 were shown in Figure 3. When sauchinone concentrations were adjusted until 200 μM, IC_50_ values of Ugt1a1-catalyzed β-estradiol, Ugt1a3-catalyzed CDCA, Ugt1a6-catalyzed serotonin, Ugt1a9-catalyzed propofol, and Ugt2b7-catalyzed zidovudine (152, 79.9, 29.8, and 23.0 μM, respectively) in MLM. Also, sauchinone showed negligible inhibitory effects on Ugt1a6 in our conditions.

### 2.4. Pharmacokinetic Study of Zidovudine with or without Sauchinone

**Figure 4:**
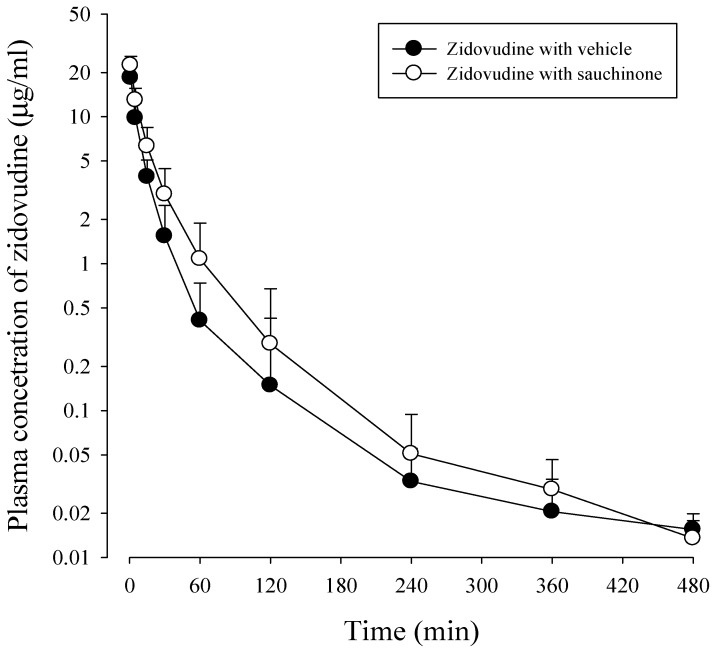
Plasma concentrations of zidovudine after intravenous administration of 15 mg/kg zidovudine with (○: *n* = 11) and without (●: *n* = 11) oral administration of 100 mg/kg sauchinone.

After administration of zidovudine together with sauchinone to mice, mean arterial plasma concentration–time profiles of zidovudine were obtained. Results are shown in Figure 4 and their relevant pharmacokinetic parameters are listed in Table 2. AUC_480 min_, CL, and CL_NR_ of zidovudine with sauchinone were increased by 53.1%, and decreased by 29.3% and 29.6%, respectively, compared to those without sauchinone, indicating that sauchinone inhibited the metabolism of zidovudine via Ugt2b7.

AUC_480 min_, the area under the plasma concentration–time curve from time zero to last sampling time, 480 min; AUC, the area under the plasma concentration–time curve from time zero to infinity; t_1/2_, terminal half-life; CL, total body clearance; CL_R_, renal clearance; CL_NR_, non-renal clearance, MRT, mean residence time; Vss, the apparent volume of distribution, Ae_0–24 h_, percentage of zidovudine excreted into urine for 24 h, GI_24 h_, percentage of zidovudine remaining or excreted in GI at 24 h.

## 3. Discussion

Inhibition of drug-metabolic enzymes in drug combination therapies is considered an important origin of adverse effects. It can lead to withdrawal of several approved drugs from the markets, causing clinical problems and economic losses [7,26]. Along with the upsurge of herb–drug combinations, inhibition of herbs towards drug-metabolic enzymes in HDIs has been raised as an important reason that limits clinical applications of herbs and drugs [17,27]. Currently, evaluating the inhibition of herbs toward UGT is recommended for safe use of herbs [16,17,18]. On the other hand, when a substrate is mainly metabolized through glucuronidation, significant increase of drug exposure via UGT inhibition has been reported (e.g., zidovudine and lamotrigine) [6,28,29], suggesting that glucuronidation inhibition can be clinically significant. Moreover, when herbs and drugs are catalyzed through the same enzymatic pathway, UGTs involved in the metabolism of herbs have the potential to inhibit drug glucuronidation [18].

As shown in Table 1, sauchinone inhibited activities of UGTs in HLM, showing the following inhibition order: UGT2B7 >> 1A6 > 1A1 > 1A3. There was negligible change in metabolic rate with increasing sauchinone concentrations up to 200 μM (our unpublished data). This could be due to un-saturation of the enzyme at the incubation condition used in this study. To explore how UGTs interacted with their substrates and inhibitors, inhibition kinetic studies of sauchinone on UGT1A6 and 2B7 were performed. As shown in Figure 2, sauchinone inhibited UGT1A6 and 2B7 in non-competitive manners. Non-competitive inhibitions of sauchinone on UGT1A6 and 2B7 indicated that sauchinone might bind different sites of substrates. In other words, sauchinone might be able to reduce UGT1A6 and 2B7 activities by binding to allosteric site of UGT without interfering with the binding of a substrate to the active site of UGT. 

Evaluating the inhibitory effect of herbs on UGTs in in vitro systems has been used to predict metabolic elimination of co-administered drugs, leading to changes in efficacy and toxicity [9,30]. However, in vitro results referring the inhibitory effects of herbs based on IC_50_ and *K*_i_ values are not sufficient to provide relevance to in vivo results especially at clinical levels. This is because more various metabolic pathways and other elimination pathways except metabolism (e.g., urinary excretion or biliary excretion) can be involved in HDIs in vivo [26,31,32]. For example, discrepancies of HDI for milk thistle, garlic extract, *Panax quinquefolius*, and *Panax ginseng* between in vitro and clinical results have been reported [18,31,33,34,35]. Investigation of HDIs in humans can accurately explain changes in efficacy or adverse reactions of co-administered drugs. However, initial evaluation of HDIs at a clinical level has been a concern due to serious problems with HDI. Hence, prediction of HDIs at preclinical level has been utilized as basic evidence to provide the potential of HDIs and its underlying mechanisms along with in vitro results [18,26].

To predict the magnitude of HDI as described in drug-drug interactions, the most common equation for HDI prediction is based on in vivo AUC alternation of a drug as a substrate and in vitro *K*_i_ and in vivo concentration of herb as an inhibitor as previously reported [26,31,36]. Parameters used to explain in vivo and in vitro inhibition potency should dictate the likelihood of pharmacokinetic drug interactions as Equation (1)

(1) $$\frac{{\mathrm{AUC}}_{i}}{{\;\mathrm{AUC}}_{0}}=1+\frac{\left[I\right]}{K_{i}}$$

Assuming that a drug is a substrate and a herb is an inhibitor toward hepatic metabolic enzymes in HDIs, the following terms are defined: AUC_i_/AUC_0_ is the predicted ratio of in vivo exposure of a drug with a herb (AUC_i_) versus that in control situation (without a herb; AUC_0_), [I] is the concentration of an herb in the liver (as an enzyme active site), and *K*_i_ is an inhibition constant of a herb. This calculation also assumes that metabolic enzyme contributes 100% of a drug metabolism. To determine whether metabolic inhibition in liver can occurs or not, the ratio of [I] to *K*_i_ is calculated because sufficient concentration of an inhibitor in the liver is a critical factor that causes metabolic inhibition. Occasionally, C_max_ has been used instead of [I], assuming that systemic exposure and tissue exposure of an inhibitor are similar [37]. In this case, [I] represents the mean steady-state C_max_ following administration of the highest proposed clinical dose [26,36]. Unfortunately, sauchinone-drug interaction cannot be estimated in human levels at this time, because hepatic concentration or C_max_ of sauchinone in human has not been reported yet.

With such situation, pharmacokinetic changes of a drug metabolized via Ugt2b7 when co-administered with sauchinone were evaluated in mice. As shown in Figure 3, sauchinone inhibited Ugt2b7 > 1a9 > 1a6 in MLM. This result suggested that Ugt2b7 inhibition by sauchinone might affect UGT2B7-mediated metabolism of drugs such as zidovudine [32,38,39]. Among clinically relevant drug interactions involving UGTs, UGT2B7 is the most commonly implicated enzyme [40], contributing to glucuronidation of drugs and endogenous compounds (i.e., bile acids, fatty acids, and steroids) [41]. Zidovudine has an especially narrow therapeutic index. Slight alternation of zidovudine concentration or unexpected increase of zidovudine exposure by UGT2B7 inhibition can cause toxicity (e.g., bone marrow toxicity or genotoxicity) [39,42]. Thus, it is meaningful to predict the potential of sauchinone–zidovudine interaction through UGT2B7. To predict the inhibitory effect of sauchinone on UGT2B7-mediated zidovudine metabolism, the following values were adjusted in Equation (1): AUC_i_/AUC of zidovudine was 1.52 from Table 2, C_max_ of sauchinone in mice plasma after oral administration of 100 mg/kg sauchinone was 1.53 μM [24] instead of [I], and *K*_i_ of sauchinone for Ugt2b7 in mouse liver microsomes was 3.34 μM (our unpublished data). U.S. FDA (2012) recommends clinical evaluation of sensitive substrate if AUC_i_/AUC of a substrate is above 1. Also ‘0.1 < [I]/*K*_i_ < 1’ is considered to have median possibility of drug interaction based on [I]/*K*_i_ standard [7]. Thus, 1.52 for AUC_i_/AUC of zidovudine and 0.458 for [I]/*K*_i_ indicated inhibitory effect of sauchinone on UGT2B7 involved zidovudine metabolism. Also the slower CL and CL_NR_ of zidovudine with sauchinone indicted that sauchinone inhibited metabolic clearance of zidovudine compared to those without sauchinone. Interestingly, except for the metabolism of zidovudine, other pharmacokinetic profiles of zidovudine were not changed by co-administration of sauchinone. Distribution and excretion of zidovudine were not affected by sauchinone, supported by comparable Vss, t_1/2_, MRT, Ae_0–24 h_, and GI_24 h_ of zidovudine between with and without sauchinone.

There are some limitations to our calculations. First, inhibition of sauchinone or zidovudine metabolites towards UGTs was not investigated. Glucuronidation, oxidation, methylation, and dehydrogenation are involved in sauchionone metabolism [24]. Glucuronindation and reduction contribute to zidovudine metabolism [43]. UGT-mediated metabolic pathway of sauchinone or zidovudine can cause auto-inhibition in the process of their own metabolism as incubation time goes on. In other words, metabolites of sauchinone or zidovudine can inhibit UGTs. Similarly, it has been reported that gemfibrozil inhibits repaglinide glucuronidation via UGT1A1, because gemfibrozil and gemfibrozil glucuronide (a main metabolite of gemfibrozil) inhibit UGT1A1 together [30]. Second, in vitro and in vivo differences both explain pharmacokinetic interactions. Substrate, inhibitor, and glucuronidated metabolite(s) can be eliminated in in vivo. However, this does not occur in an in vitro microsomal incubation system. When the accumulation of metabolites happens in the reaction process, it inhibits enzyme activity [6]. Also, several other factors such as protein binding, active uptake, and efflux transporters in tissues may affect the estimation of unbound drug concentrations at interaction site. In this respect, in vitro data tend to underestimate inhibition of drug glucuronidation compared to in vivo [44]. Moreover, renal clearance and cytochrome P450-mediated metabolism can be altered by sauchinone in vivo, which can affect the elimination route of zidovudine [43]. In addition, individual difference in intestinal bacteria might influence sauchinone absorption in the blood that might influence in vivo extrapolation results [43]. Thus, extrapolation from in vitro data to in vivo drug interaction should be taken with caution.

## 4. Materials and Methods

### 4.1. Chemicals and Reagents

Sauchinone was extracted and purified according to a method reported previously [20]. The purity of sauchinone was determined to be over 98% by using Waters Acquity™ Ultra Performance LC system (Waters Corp., Milford, MA, USA) equipped with an ACQUITY UPLC^®^BEH C18 column (2.1 × 150 mm, 1.7 μm) and detailed information for structure elucidation and purity experiment were provided in supplementary data. Pooled human liver microsomes from a mixed pool of 50 donors (HLMs; BD Ultra Pool HLM 50, cat. 452156) and human cDNA-expressed UGT enzymes (UGT1A6 and 2B7) were purchased from Corning Life Sciences (Woburn, MA, USA). Alamethicin, β-estradiol, β-estradiol-3-glucuronide, chenodeoxy cholic acid, lithocholic acid, chrysin, efavirenz, niflumic acid, 1-naphthol, serotonin hydrochloride, trifluoperazine dihydrochloride, zidovudine, chenodeoxycholic acid, lithocholic acid, the reduced form of β-nicotinamide adenine dinucleotide phosphate (NADPH; as a tetrasodium salt), and uridine 5′-diphosphoglucuronic acid trisodium salt (UDPGA) were purchased from Sigma-Aldrich (St. Louis, MO, USA). Midazolam and propofol were supplied from Korea Ministry of Food and Drug Safety (Chung-cheong-buk-do, Republic of Korea). Hecogenin, propofol-*O*-glucuronide, serotonin-*O*-glucuronide, trifluoperazine-*N*-glucuronide, and zidovudine-5′-glucuronide were purchased from Tokyo Chemical Industry Co. (Tokyo, Japan). Chenodeoxy cholic acid-24-acyl-β-d-glucuronide was purchased from Carbosynth (Berkshire, UK). Carbamazepine [an internal standard (IS) of ultra-performance liquid chromatography-tandem mass spectrometry (UPLC-MS/MS)] was purchased from Wako Co. (Tokyo, Japan). All other chemicals and reagents used were of analytical grade.

### 4.2. Animals

To conduct in vitro (mice liver microsomal study) and in vivo (pharmacokinetic study) investigations using mice, animal studies were approved by the Institute of Laboratory Animal Resources of Dongguk University, Seoul, Republic of Korea (IACUC-2016-046). Five-week-old (20–25 g) male Institute of Cancer Research mice were purchased from the Charles River Company of Korea (Orient, Seoul, Republic of Korea). Upon arrival, mice were randomized and housed at five mice per cage under strictly controlled environmental conditions (22–25 °C and 48–52% relative humidity) with a 12 h light/dark cycle at 150–300 lx luminous intensity. All mice were provided food and water and were maintained during this study.

### 4.3. Inhibitory Effects of Sauchinone on UGT Activities

Inhibitory effects of sauchinone on UGT1A1, 1A3, 1A4, 1A6, 1A9, and 2B7 activities were evaluated using HLMs. Formations of β-estradiol-3-glucuronide, chenodeoxy cholic acid-24-acyl-β-d-glucuronide, trifluoperazine-*N*-glucuronide, serotonin-*O*-glucuronide, propofol-*O*-glucuronide, and zidovudine-5′-glucuronide represented glucuronidation efficacy of UGT1A1, 1A3, 1A4, 1A6, 1A9, and 2B7, respectively, in HLMs. As UGT selective substrates, β-estradiol, chenodeoxy cholic acid, propofol, and zidovudine were dissolved in methanol, whereas trifluoperazine and serotonin were dissolved in 50% ethanol. These dissolved substrate solutions were serially diluted to required concentrations as a UGT cocktail set containing β-estradiol, chenodeoxy cholic acid, trifluoperazine, serotonin, propofol, and zidovudine. Spiked volume of UGT cocktail set was 1% (*v/v*) of total volume of mixture. Concentrations of UGT selective substrates were used close to their reported K_m_ values [9]: 10 μM β-estradiol, 15 μM chenodeoxy cholic acid, 40 μM trifluoperazine, 40 μM serotonin, 100 μM propofol, and 100 μM zidovudine as substrate of UGT1A1, 1A3, 1A4, 1A6, 1A9, and 2B7, respectively. The reaction mixture containing 0.25 mg/mL protein of HLM, 100 mM Tris buffer (pH 7.6), 5 mM MgCl_2_, 25 μg/mL of alamethicin, the UGT selective substrates’ cocktail set and various concentrations of sauchinone (0–200 μM) or a well-known inhibitor of each UGT isoform were pre-incubated for 30 min on ice to allow formation of alamethicin pores. The reaction was initiated by adding 5 mM UDPGA to a final volume of 150 μL and incubated at 37 °C for 15 or 60 min in a thermomixer at 500 rpm. Each reaction was stopped by adding 150 μL of ice-cold methanol containing 50 ng/mL IS. These mixtures were then centrifuged (12,000 rpm for 10 min at 4 °C) and 5 μL of the supernatant was injected into a UPLC-MS/MS system. As positive controls, well-known inhibitors such as chrysin, lithocholic acid, hecogenin, 1-naphthol, niflumic acid, and efavirenz for UGT1A1, 1A3, 1A4, 1A6, 1A9, and 2B7, respectively, were used to validate the experiments. Their IC_50_ values were compared to that of sauchinone. All inhibitors were dissolved in methanol (except for hecogenin, which was dissolved in dimethyl sulfoxide) and serially diluted to the required concentrations using the same solutions. The final concentration of organic solvent of inhibitors was 1% (*v/v*). All studies were performed in triplicate and mean values were used in the analysis. The half maximal inhibition constant (IC_50_) of sauchinone on UGT isoforms was estimated using GraphPad program.

### 4.4. Inhibition Constant (K_i_) of Sauchinone on UGT1A6 and 2B7-Mediated Glucuronidation Activities

Based on *IC*_50_ values, *K_i_* values of sauchinone for UGT1A6 and 2B7 were determined. Concentrations of 0–200 μM of sauchinone were used. Other procedures were similar to those mentioned above in the study of reversible inhibitory effects of sauchinone. Inhibitory characteristics of sauchinone were initially estimated by nonlinear least squares regression analysis and *K_i_* values were determined by Dixon plots [45].

### 4.5. Inhibitory Effects of Sauchinone on UGT Activities Using MLM

Inhibitory effects of sauchinone on UGT1A1, 1A3, 1A6, 1A9, and 2B7 activities were evaluated using MLM. Mice were sacrificed and then livers were removed and rinsed with saline. After weighing the livers, they were homogenized with a four-fold volume of ice-cold homogenizing buffer (0.154 M KCl, 50 mM Tris-base, 1 mM EDTA, pH 7.4). After centrifugation for 35 min at 9000× *g* and 4 °C, the supernatants were centrifuged for 95 min at 100,000× *g* and at 4 °C. The supernatants were discarded and the pellets were homogenized again with ice-cold homogenizing buffer. These aliquots were stored at −80 °C. The subsequent process to evaluate the inhibitory effects of sauchinone on UGT activities in MLM was the same as those using HLM.

### 4.6. UPLC-MS/MS Analysis for Metabolites of UGTs Substrates

Metabolites of UGT-selective substrates were detected using an Waters UPLC-XEVO TQ system (Waters Corporation, Milford, MA, USA) in multiple reaction monitoring (MRM) mode with an ESI interface for positive ions ([M + H]^+^) and negative ions ([M − H]^−^). Separation was performed on a reversed-phase C_18_ column (BEH, 1.7 × 100 mm i.d., 2.1 μm particle size; Waters, Dublin, Ireland) maintained at 30 °C. The mobile phase consisted of water containing 0.1% formic acid (A) and acetonitrile (B) at a ratio of 85:15 (*v/v*) for 3 min and gradually changed to 10:90 (*v/v*) for 5.5 min. This composition was maintained until 6.5 min and then back to initially composition at a flow rate of 0.3 mL/min. The total run time was 9 min. 

Turbo ion-spray interface was operated in positive ion mode at an ion capillary voltage of 2500 V and a temperature of 350 °C. Operating conditions (gas flow, 650 L/h; cone gas flow, 10 L/h) were optimized by flow injection of a mixture of all analytes. The *m*/*z* value (CE value) for each metabolite of UGT-selective substrate was as follows: β-estradiol-3-glucuronide (*m*/*z* 446.93→270.90, −40 V of CE), chenodeoxy cholic acid-24-acyl-β-d-glucuronide (*m*/*z* 567.33→567.33, −10 V), trifluoperazine-*N*-glucuronide (*m*/*z* 584.23→408.20, 25 V), serotonin-*O*-glucuronide (*m*/*z* 353.13→177.10, 10 V), propofol-*O*-glucuronide (*m*/*z* 353.13→177.10, −19 V), and zidovudine-5′-glucuronide (*m*/*z* 442.13→125.0, −26 V).

### 4.7. Data Analysis

UGT-mediated activities in the presence of sauchinone as an inhibitor were expressed as percentages of corresponding control values (in absence of sauchinone). From percentages of control activity versus inhibitor concentrations, a sigmoid shaped curve was fitted to the data and IC_50_ as an enzyme inhibition parameter was calculated by fitting the Hill equation to the data using GraphPad Prism 5 (GraphPad Software Inc., San Diego, CA, USA).

### 4.8. Pharmacokinetic Study of Zidovudine with or without Sauchinone

To investigate the inhibitory effects of sauchinone on UGT2B7-mediated metabolism, zidovudine was chosen as an example drug mainly metabolized via UGT2B7 [46]. Surgical procedures of mice were conducted under tiletamine HCl and zolazepam HCl anesthesia by intramuscular injection. Jugular vein (for zidovudine administration) and carotid artery (for blood sampling) cannulations were carried out using catheters (BASi, West Lafayette, IN, USA). Five hours after surgery, drug administration and blood sampling were allowed [24]. Thirty minutes after oral administration of 100 mg/kg sauchinone, 15 mg/kg of zidovudine was administered intravenously to mice. Blood samples were collected via the carotid artery at 0, 1, 5, 15, 30, 60, 120, 240, 360, and 480 min after administration of zidovudine. A 10-μL of blood was collected into a micro-vial with 50-μL of 12.5 units/mL heparinized saline using micro-sampling system. After centrifugation of each micro-vial, a 50-μL of plasma with heparinized-saline was collected from the supernatant, which was analyzed by UPLC-MS/MS. Also, at the end of 24 h, each metabolic cage was flushed with 5 mL of distilled water and urine samples were collected (Ae_0–24 h_). The gastrointestinal tract was cut and extracted with 20 mL of methanol, of which supernatant was collected (GI_24 h_). Urine and GI samples were then analyzed using UPLC-MS/MS.

After the analysis of zidovudine concentrations in plasma by UPLC-MS/MS, pharmacokinetic parameters were calculated as follows. The area under the plasma concentration–time curve from time zero to the last measured time to infinity or last sampling time (480 min) (AUC or AUC_480 min_) was calculated using the trapezoidal rule method. Standard methods [47] were used to calculate pharmacokinetic parameters using a non-compartmental analysis (WinNonlin 2.1; Pharmasight Corp., Mountain View, CA, USA). Peak plasma concentration (C_max_) and time to reach C_max_ (T_max_) were read reversibly from the extrapolated data. 

## 5. Conclusions

The present study investigated the inhibitory potential of sauchinone towards UGTs to cause drug interactions for the first time. In detail, sauchinone inhibited UGT activities in the following order: UGT2B7 > 1A6 > 1A1. It noncompetitively inhibited UGT1A6 and 2B7 activities in vitro. Sauchinone also increased systemic exposure of zidovudine, a substrate of UGT2B7, through UGT2B7 inhibition in vivo mice. All these results provide an early warning for the combination between sauchinone and drugs mainly metabolized by UGT2B7. Due to various contributions of UGT isoforms toward drug metabolism as well as species and individual differences, further clinical investigations should be considered.

## Figures and Tables

**Table 1:** IC_50_ (μM) values of sauchinone (*n* = 3 for each UGT isoform) and well-known inhibitors (*n* = 1 for each UGT isoform) for inhibition of various UGTs in human liver microsomes. Data were expressed as the mean values. The minimum and maximum values of IC_50_ were expressed in the parentheses.

UGTs	Sauchinone	Well-Known Inhibitor	
UGT1A1	8.83 (5.40–14.5)	Chrysin	28.3
UGT1A3	43.9 (18.9–102)	Lithocholic acid	69.8
UGT1A4	391 (253–603)	Hecogenin	3.99
UGT1A6	0.758 (0.383–1.50)	1-Naphthol	35.2
UGT1A9	919 (517–1632)	Niflumic acid	0.755
UGT2B7	0.279 (0.224–0.347)	Efavirenz	75.4

**Table 2:** Means (± standard deviations) of pharmacokinetic parameters of zidovudine after intravenous administration of 15 mg/kg zidovudine with or without oral administration of 100 mg/kg sauchinone (*n* = 11 for each group).

Parameters	With Vehicle	With Sauchinone
Body weight (g)	30.6 ± 3.74	31.7 ± 3.37
AUC_480 min_ (μg min/mL)	227 ± 61.9	344 ± 142 ^a^
AUC (μg min/mL)	228 ± 60.8	349 ± 140 ^a^
t_1/2_ (min)	207 ± 133	178 ± 93.1
CL (mL/min/kg)	68.7 ± 18.7	48.6 ± 16.1 ^a^
CL_R_ (mL/min/kg)	34.0 ± 11.2	24.0 ± 8.02 ^a^
CL_NR_ (mL/min/kg)	34.8 ± 10.5	24.5 ± 9.31 ^a^
MRT (min)	55.8 ± 28.2	43.5 ± 16.2
Vss (mL/kg)	3307 ± 1670	2105 ± 1229
Ae_0–24 h_ (% of dose)	50.3 ± 8.01	50.2 ± 8.41
GI_24 h_ (% of dose)	0.672 ± 0.999	0.321 ± 0.646

^a^ Significantly different (*p* < 0.05) from with vehicle.

## Supplementary Materials

Supplementary materials are available online.
