# Supplementary material for: Inhibitory Effect of Sauchinone on UDP-Glucuronosyltransferase (UGT) 2B7 Activity
Source: Molecules. 2018 Feb 9;23(2):366. doi: 10.3390/molecules23020366 (PMC6017115; doi:10.3390/molecules23020366)
Supplement: Supplementary file 1 [file molecules-23-00366-s001.pdf]

Mass spectrum of Sauchinone

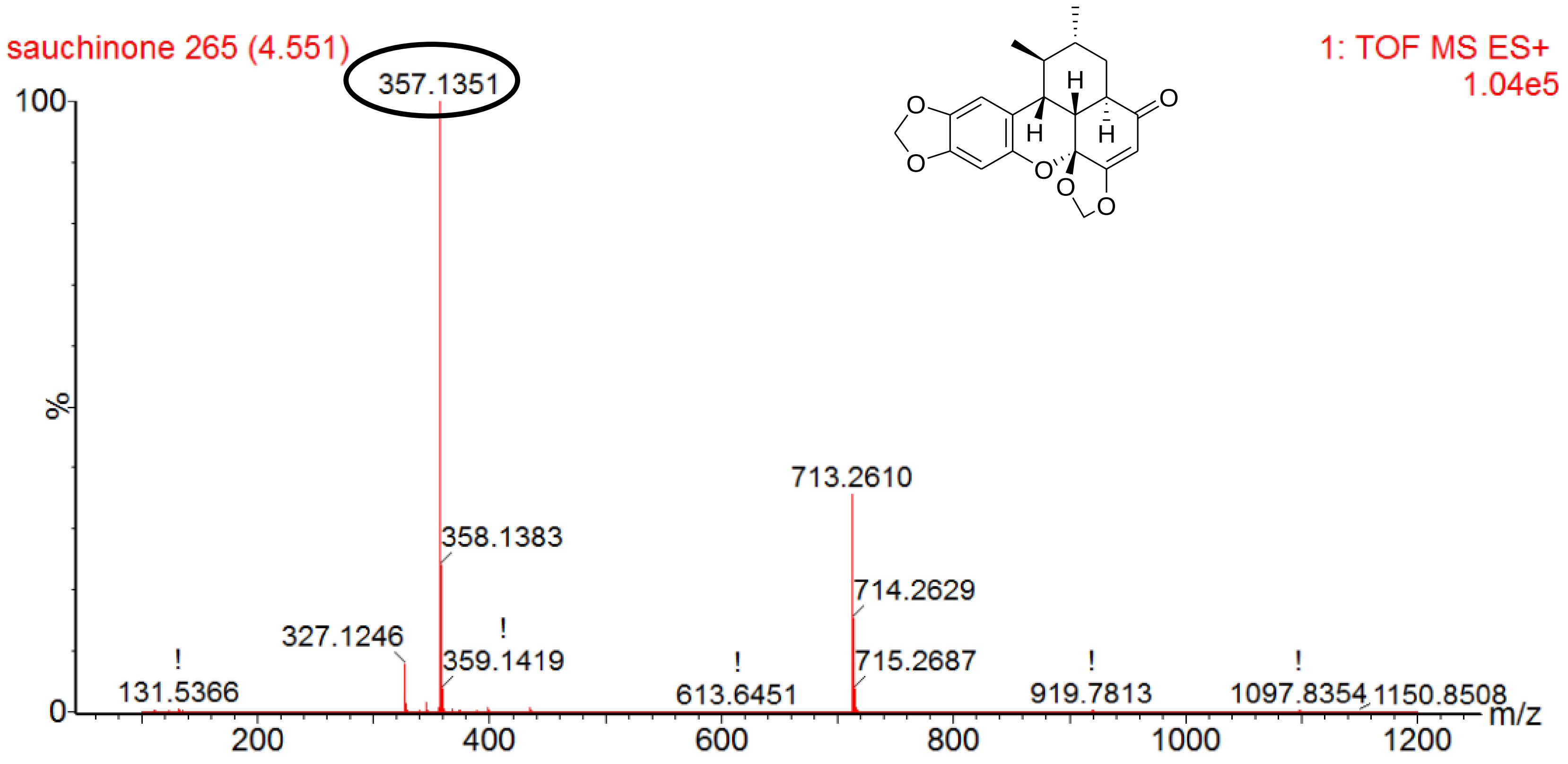

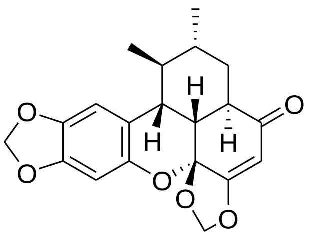

Elemental Composition Report

Single Mass Analysis

Tolerance = 100.0 PPM / DBE: min = -1.5, max = 50.0
Element prediction: Off
Number of isotope peaks used for i-FIT = 3

Monoisotopic Mass, Even Electron Ions
71 formula(e) evaluated with 10 results within limits (up to 100 closest results for each mass)
Elements Used:
C: 0-100 H: 0-100 O: 0-50
sauchinone 265 (4.551)
1: TOF MS ES+

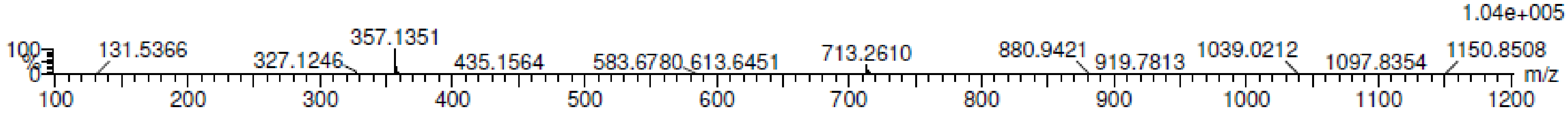

Minimum:				-1.5				
Maximum:		5.0	100.0	50.0				
Mass	Calc. Mass	mDa	PPM	DBE	i-FIT	Norm	Conf(%)	Formula
357.1351	357.1338	1.3	3.6	10.5	321.5	2.882	5.60	C20 H21 O6
	357.1397	-4.6	-12.9	1.5	330.3	11.675	0.00	C13 H25 O11
	357.1279	7.2	20.2	19.5	323.0	4.320	1.33	C27 H17 O
	357.1491	-14.0	-39.2	14.5	320.8	2.164	11.48	C24 H21 O3
	357.1186	16.5	46.2	6.5	327.9	9.222	0.01	C16 H21 O9
	357.1549	-19.8	-55.4	5.5	327.1	8.516	0.02	C17 H25 O8
	357.1127	22.4	62.7	15.5	319.0	0.370	69.08	C23 H17 O4
	357.1643	-29.2	-81.8	18.5	326.0	7.360	0.06	C28 H21
	357.1033	31.8	89.0	2.5	331.3	12.681	0.00	C12 H21 O12
	357.1702	-35.1	-98.3	9.5	320.7	2.087	12.41	C21 H25 O5

^{1}H NMR spectrum of Sauchinone in $CDCl_3$(400MHz)

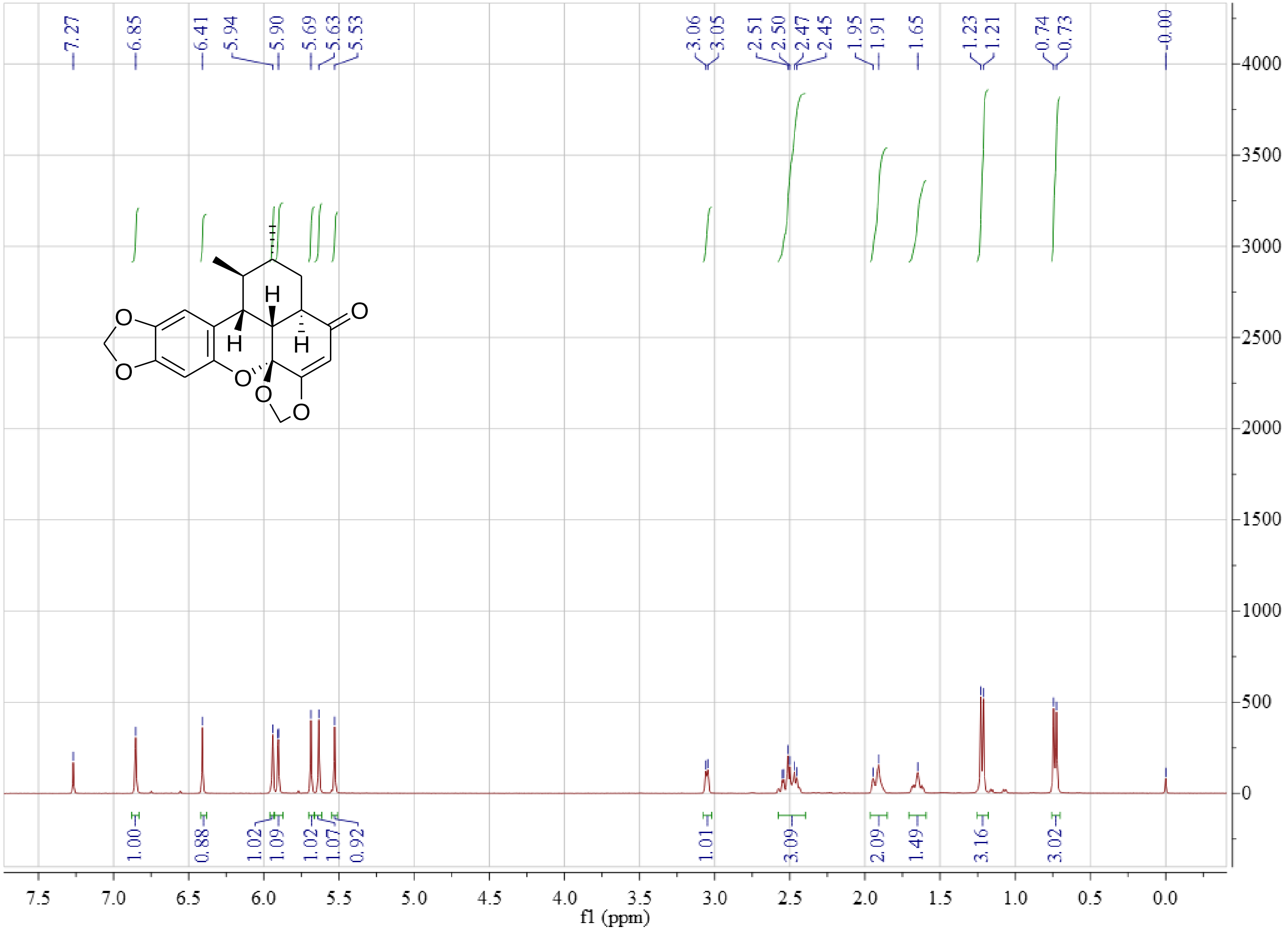

^{13}C NMR spectrum of Sauchinone in $CDCl_3$(100MHz)

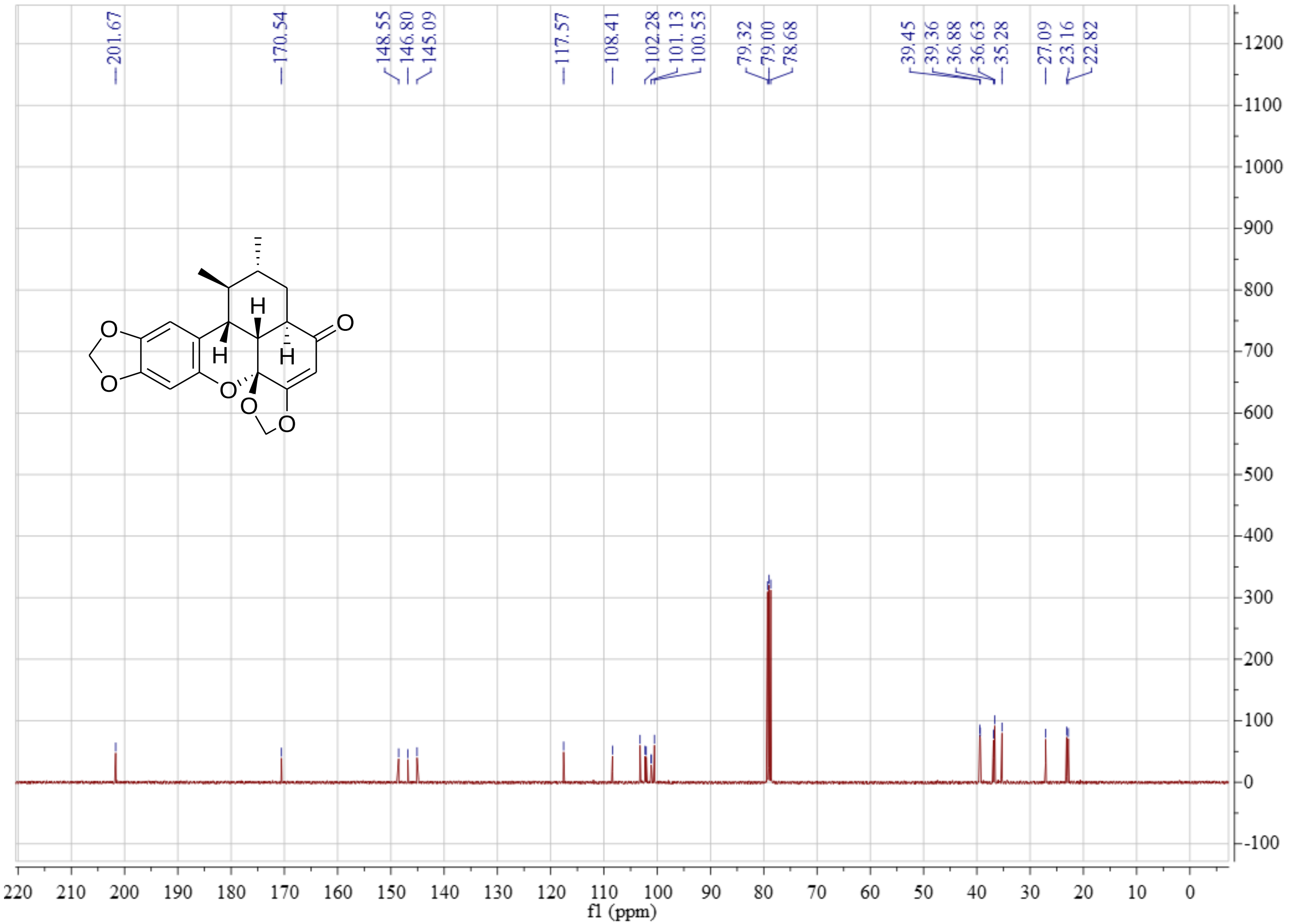

Peak area value of Sauchinone and Blank

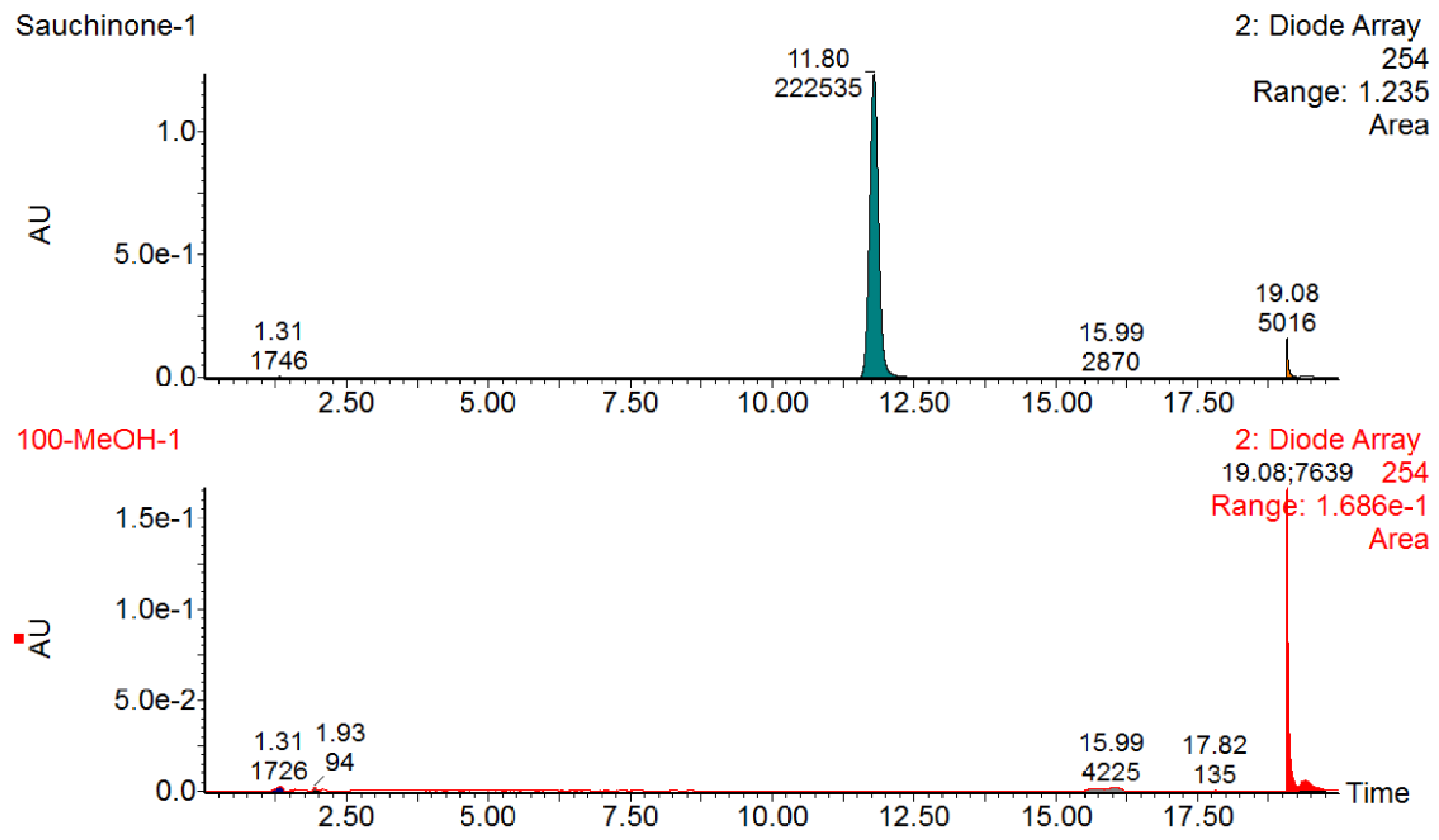

Analytical Condition for Sauchinone

- UPLC: Waters Acquity™ Ultra Performance LC system (Waters Corp., Milford, MA)
- Column: ACQUITY UPLC®BEH C18 column (2.1 mm×150 mm, 1.7 μm)
- Mobile phase

A (0.1% formic acid in water)

B (0.1% formic acid in acetonitrile)

Injection volume: 2 μl (1 mg/ml in 100% MeOH)

Gradient elution program of UPLC

Time (min)	Flow rate (ml/min)	%A	%B
0.0-14.0	0.3 ml/min	60	40
14.0-14.5	0.3 ml/min	0	100
14.5-17.0	0.3 ml/min	0	100
17.0-17.5	0.3 ml/min	60	40
17.5-20.0	0.3 ml/min	60	40
